# Predicting the Compressive Properties of Carbon Foam Using Artificial Neural Networks

**DOI:** 10.3390/ma18112516

**Published:** 2025-05-27

**Authors:** Debela N. Gurmu, Krzysztof Wacławiak, Hirpa G. Lemu

**Affiliations:** 1 Faculty of Materials Engineering, Silesian University of Technology, 40-019 Katowice, Poland; debela.negasa.gurmu@polsl.pl (D.N.G.); krzysztof.waclawiak@polsl.pl (K.W.); 2 Faculty of Science and Technology, University of Stavanger, N-4306 Stavanger, Norway

**Keywords:** cellular solid, carbon foam, artificial neural network, property prediction

## Abstract

This article focusses on predicting the compressive properties of polyurethane-derived carbon foam using an artificial neural network (ANN) approach. To train the model, strain, pore density (20, 40, and 60 ppi), and solvents (acetone, ethanol, and methanol) were used as inputs, while compressive stress was used as output. Categorical variables like acetone, ethanol, and methanol were converted to binary form before training the ANN model by using one-hot encoding mechanism. Both inputs and outputs were normalized to prevent features with larger numerical ranges from dominating the training process. A feed-forward ANN with four hidden layers, each containing 100 neurons, was constructed. The performance of the ANN model was tested using three metrics: mean square error (MSE), mean absolute error (MAE), and coefficient of determination (R^2^). The Adam optimizer was used to optimize the weights and biases of the ANN. The model was trained for 10,000 epochs with a batch size of 50. Rectified Linear Unit (ReLU) and linear functions were used as activation functions for the hidden layers and the output layer, respectively. From the results, overall average MSE, MAE, RMSE, and R^2^ values of 36.34, 4.42, 6.00, and 0.9797, respectively, were obtained.

## 1. Introduction

Carbon materials can be designed into different dimensions, including carbon quantum dots, carbon nanotubes, graphene, carbon fiber, and foam-structured carbon materials [1]. Among these, carbon foams (CFs) have received the greatest attention due to their lightweight, controllable thermal conductivity, high specific strength, high-temperature tolerance (up to 3000 °C in inert atmosphere), abundant source, and low cost [2,3]. Carbon foam is mainly synthesized from polymer precursors like polyurethane and melamine foams, mesophase pitch, and coal tar pitch [4]. Due to its excellent electrical and thermal properties, CF has applications in various fields, including energy storage, thermal insulation, and lightweight structural components, making it a versatile material for three-dimensional structural applications [5,6].

Researchers have used many methods to investigate the properties of cellular solids. Most of the models available in the literature are either micro-mechanical or use complex mathematical relationships to predict the behavior of cellular solids. For instance, the Gibson model is a commonly used model based on a micro-mechanical model (cubic array unit cell), where deformation is controlled by bending of individual struts [7], while the Avalle model is based on macro-mechanical parameters [8]. The micro-mechanical analysis focuses on individual cells and struts, and it requires detailed knowledge of cell geometry, cell topology, and material properties. However, this model could be quite complex to manage because of its need for at least a rough analysis of the foam structure [9].

Additionally, the micro-mechanical model assumes that the cellular solid is a regular array of cells like cube [10], truncated cube [11], rhombic dodecahedron [12], diamond unit cells [13], and tetrakaidekahedral/kelvin unit cell [14]. It has been pointed out that micro-mechanics/unit cell-based models can provide important results; however, they are significantly limited by their inability to account for inherent microstructural imperfections like irregular cell shape and size, thickness variation in cell walls, non-uniform solid distribution in cell walls, and curved/corrugated cell walls which are common in most real cellular materials, whose cell structures are in practice non-periodic, non-uniform, and disordered [15]. In contrast, the macro-mechanical model focuses on the bulk behavior of the material and is simpler, with fewer computational resources needed. Additionally, the macro-mechanical model does not require the micro-structural features of the foam to be modeled. However, this modeling technique is difficult to employ when investigating the effect of micro-structural features on the foam’s overall behavior [16].

Both micro-mechanical and macro-mechanical models can be analyzed using either Euler–Bernoulli or Timoshenko beam theory [17]. While Euler–Bernoulli beam theory ignores the effect of shear deformation, Timoshenko beam theory takes shear deformation and rotational inertia effects into account in addition to Euler–Bernoulli beam theory, which only considers the effect of bending, twisting, and axial deformation [13,18]. In order to consider the effect of micro-structural imperfections, researchers currently employ an imaged-based analysis model (micro-tomography scan) that captures the real structure of cellular solids like local density, cell size, shape distribution, and cell wall thicknesses [19]. It provides geometries much closer to the real foam structure and has advantages including high resolution, three-dimensional imaging, and multiscale imaging from nanoscale to microscale. In contrast, this method is difficult to employ in geometry optimization as it cannot avoid artifacts, and not all materials are suitable for computed tomography scan (CT scan) [20].

Other models used to predict the properties of cellular solids are phenomenological models like the Rusch model, which only needs simple fitting of experimental data to understand mechanical behavior without direct relationship with the physics of the phenomenon [21]. However, this approach does not consider the effect of density at all, and it does not provide an explanation for the micro-structural deformation and failure mechanisms of the foam material, such as its weak spots, stress concentrations, and localized deformations. Furthermore, this model cannot describe the stress–strain relationship at high strain rates (10^5^ s^−1^) or under explosive loading, and the densification region fits poorly at a relatively high density (low void porosity) [22].

One of the most practical ways to address this issue is to employ finite element analysis (FEA), which is a powerful tool to simulate the mechanical properties of cellular materials. This is because FEA approaches are widely used for simulating the mechanical behavior of cellular materials due to their exceptional flexibility in modeling complex material and structural behaviors [23]. However, the high cost of numerical analysis tools also presents other challenges in using this method. For this reason, researchers propose artificial neural networks (ANNs), which promise new methods with low cost and high accuracy to predict the properties of any structure and material regardless of its shape and structure.

ANNs are one of the most popular algorithms of machine learning algorithms and are widely used tools in various research disciplines [24,25]. They can accurately predict complex non-linear relationships between input and output of the system without the need for explicit model equations [26]. In addition, an ANN is considered as an effective alternative to traditional statistical techniques for function approximation and data fitting since it does not need a hypothetical premise concerning the mathematical models [27]. In the field of cellular solids, ANNs have become highly effective tools for modeling, optimizing, and predicting material properties. Lightweight materials such as carbon foam are characterized by their cellular structures and display complex mechanical and thermal behaviors due to their intricate microarchitecture. Using ANNs, researchers have estimated key properties such as the thermal analysis of a heat exchanger [28,29], mechanical properties [30], thermal conductivity [31], energy consumption [32], and energy absorption [33,34] based on material geometry and composition. Additionally, an ANN supports inverse design, facilitating the development of cellular structures tailored to specific needs. This integration of material science and computational intelligence has significantly advanced the creation of cutting-edge cellular materials for applications in aerospace, biomedical, and automotive fields [35].

Traditional approaches, such as support vector regression (SVR), Gaussian process regression (GPR), and other empirical models, often fail to capture the complex and non-linear relationships that govern the behavior of cellular solids like carbon foam. The most common challenges of these come from complex multi-effect interaction and non-linear damage/failure mechanisms of cellular solids (especially brittle foam such as carbon foam). These non-linear damage/failure mechanisms of cellular solids mainly result from progressive cell wall collapse [36,37]. These difficulties limit the application of SVR, GPR, and other empirical models to easily predict the properties of cellular solids like in the case of convectional materials. This challenge motivates researchers to find alternative methods that can easily predict the mechanical properties of cellular solids without knowing the complex and non-linear multi-scale interaction among cellular solids as well as the damage mechanisms. One of the most promising and interesting approaches proposed by researchers is data-driven methods such as ANNs, which have become important tools for studying multiscale materials, estimating the mechanical responses of materials based on the characteristic information of materials, and assisting in material design [38]. ANNs are particularly useful in modeling non-linear problems, where analytical solutions are rather complex [39]. Furthermore, with the advancements in computer hardware, machine learning has evolved into a data-driven tool capable of deciphering any non-linear relationships within the data. This offers a new avenue for exploring the intricate relationships between complex structures of cellular solids and their mechanical responses [40]. As shown in the summary of review of relevant articles shown in Table 1, ANNs are the most used tools in the area.

The observation from the reviewed and listed articles indicates that there are no research works that used the effect of pore density and solvents at the same time. Additionally, a research gap in applying the ANN method to highly non-linear and brittle cellular solids is observed. Furthermore, most reported articles used metallic foam and cement foam rather than carbon foam. Thus, the aim of this article was to predict the compressive properties of polyurethane-derived carbon foam to investigate the effect of pore density and solvents using artificial neural networks.

## 2. Materials and Methods

### 2.1. Materials

**Figure 1:**
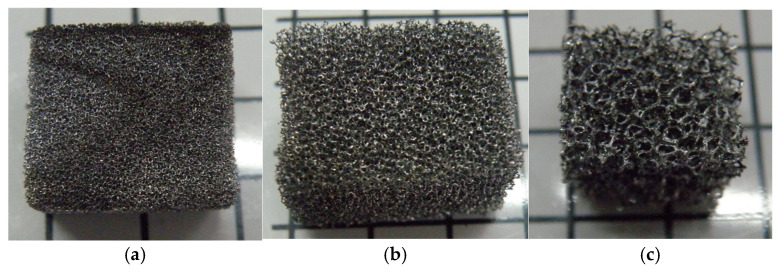
Carbon foam samples of different density: (**a**) 60, (**b**) 40, and (**c**) 20 ppi (reused from [55], an open access article distributed under the terms of the Creative Commons CC-BY license).

**Figure 2:**
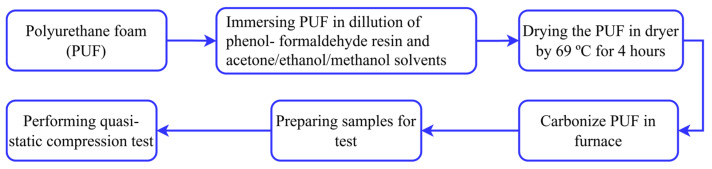
Sample preparation procedure.

In the study reported in this article, carbon foam derived from polyurethane foam was used. Pore density and solvents are two main factors that were varied in the research. Both the pore density and the solvent have three levels. Initially, polyurethane foam with different pore sizes (20, 40, and 60 ppi) was selected as the precursor and then immersed in a dilution of phenol-formaldehyde resin with different solvents (acetone, ethanol, and methanol). The purpose of adding solvents into phenol-formaldehyde is to reduce the viscosity of phenol-formaldehyde resin. This is because a higher viscosity of phenol-formaldehydes results in a closed wall carbon foam and higher non-uniform strut thickness. The purpose of covering foam with this mixture is to maintain the stability of pore size and shape, before and after carbonization process. The polyurethane foam was then dried at 69 °C for 4 h in a dryer and the samples were carbonized in the furnace at 1000 °C. Lastly, the samples with 96% carbon content in their chemical compositions with different pore sizes (Figure 1) were obtained and prepared for the uniaxial quasi-static compression test with steps shown in Figure 2. A uniaxial compression test was performed on a Zwick Roel universal testing machine (ZwickRoell GmbH & Co. KG, Ulm, Baden-Württemberg, Germany) of 2.5 kN at room temperature with a loading rate of 5 mm/min. The sample preparation and the stress–strain result for carbon foam with different pore density and different solvent was presented in a previously published paper [55].

### 2.2. Artificial Neural Networks

**Figure 3:**
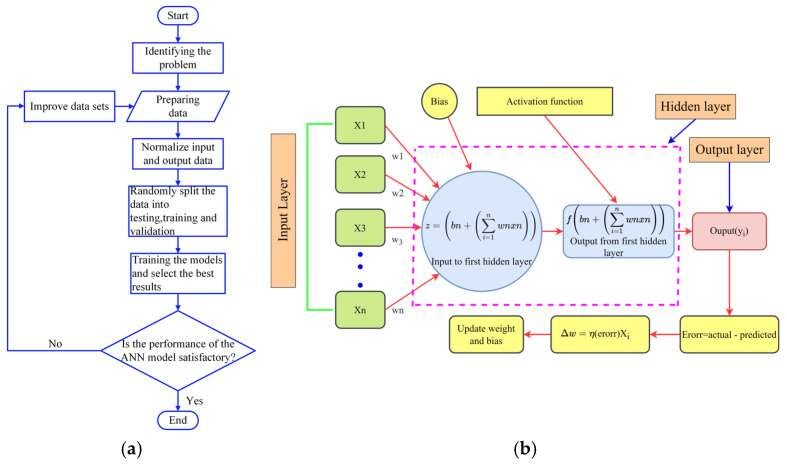
(**a**) General steps of ANN, and (**b**) architecture of ANN with input layer, hidden layer, and output layer.

ANNs are among the best methods employed in ML and used to process complex and non-linear data [56]. Additionally, an ANN is a mathematical tool inspired by the biological human nervous system [57] and it is composed of input, hidden, and output layers, and neurons, weights, and biases [58]. The input layer handles the collection of input variables, while the hidden layer functions as the central processing unit of the system. The output layer handles the system’s output [59]. Weights are the parameters that adjust the strength of the connection between neurons in different layers of the network, while biases provide flexibility to the model by allowing neurons to adjust independently of their input. The flowchart shown in Figure 3a illustrates the process used to develop and optimize the ANN models in this article. It starts with the identification of the problem, followed by the preparation of data, which includes the normalization of inputs and outputs and the division of data into test, training, and validation sets. Then, the model is trained, and the best results are selected. The process stops when the performance of the ANN is satisfactory; otherwise, the dataset is improved and the process repeats. This iterative approach ensures that the model is refined for optimal accuracy and reliability.

In this study, a feed-forwarded ANN was developed to predict the compressive stress values of carbon foam based on input parameters, including pore density, solvents, and strain. These data were obtained from a quasi-static compression test of polyurethane-derived carbon foam. The ANN model consisted of an input layer followed by hidden layers that learn complex relationships within the data, and an output layer that provides the predicted compressive stress. Each neuron in the hidden layers applies the ReLU activation function (Figure 3b) to introduce non-linearity and enhance learning capability, while the output layer uses a linear activation function to generate continuous compressive stress values. The network was trained using backpropagation, with the Adam optimization algorithm to minimize the MSE loss function. The dataset was randomly divided into training, validation, and testing sets to ensure the model generalizes well to unseen data. The model performance was evaluated using metrics such MSE, MAE, RMSE, and R^2^ to assess prediction accuracy. The details of the way the ANN was built is discussed in Section 2.2.1, Section 2.2.2, Section 2.2.3 and Section 2.2.4.

#### 2.2.1. Preparing the Training, Testing, and Validation Datasets

In this study, 22,074 data were used for training, testing and validation, which were obtained from quasistatic compression tests of polyurethane-derived carbon foam. The data were divided randomly into 70% (15,454) for training, 15% (3310) for testing, and 15% (3310) for validation. The purpose of adding a testing and validation dataset is to overcome the over-fitting problem [60], while the training process is the adjustment of weights and biases to obtain output data through applying a proper method [61]. The validation set was used to adjust the hyperparameters (epochs, learning rate, number of hidden neurons in a neural network). The validation subset was used to further check the network, confirm its accuracy, or assess its capability to predict unknown cases. The test set was used to assess the (generalization) performance of the neural network. During the random splitting of data into training, testing, and validation datasets, attention was given to avoid the imbalance across datasets because an imbalanced split of data affects model training and evaluation reliability. For instance, in our case, there were 1014, 1173, and 1123 data for 20, 40, and 60 ppi in testing datasets, respectively. Even though this difference is small compared to the size of our data, it was arranged manually to avoid the effect of data imbalance. Additionally, to prevent overriding between different numerical scales and premature saturation of hidden nodes, as well as to reduce the risk of having larger errors, the normalization of input and output data was performed using Equation (1) [62].

(1) $$\mathrm{Normalization}\;(N)=\frac{X-X_{min}}{X_{max}-X_{min}}$$

where *N*, *X*, *X_min_*, and *X_max_* are the normalized values, the serial number of the dataset, and the minimum and maximum values in the data samples, respectively.

#### 2.2.2. Construction of the ANN Model

Before constructing the ANN model, the number of hidden layers, activation functions, learning rate, number of neurons per layer (especially in hidden layers), and architecture of the ANN are the main parameters of a neural network that require attention and must be defined before starting the training [63]. Nevertheless, there are no clear theories and methods used to decide these parameters. Evaluating the number of hidden layers and the number of neurons in each hidden layer is the most challenging task in developing the ANN model. For this reason, the number of neurons in hidden layers can only be found by trial and error. In this article, one input layer with 5 neurons, four hidden layers with 100 neurons each, and one output layer with one neuron were selected. The choice of the number of hidden layers and the number of neurons in each hidden layer was initially inspired by a previously published paper [64].

Furthermore, to give a better justification for fixing the number of hidden layers as well as the number of neurons in the hidden layers, a grid search approach was used to optimize the architecture of the ANNs. Accordingly, the optimal number of hidden layers was selected from 1, 2, 3, and 4 (where a higher number of hidden layers was found to be computationally expensive). In contrast, the number of neurons in hidden layers was varied from 25 up to 200 (with step size 25). The number of epochs was varied from 100, 1000, and 10,000, and the performance of the ANNs model was compared for each combination of number of hidden layers, number of neurons in each hidden layer and the number of epochs by recording the MAE, MSE, and R^2^ values. The recorded MAE, MSE, and coefficient of determination (R^2^) for comparison are shown in Table 2.

**Figure 4:**
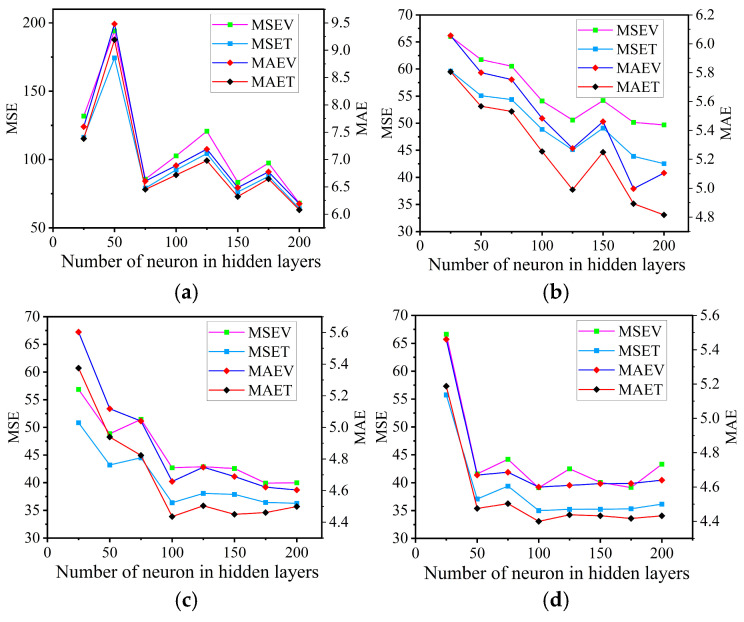
Variation in MSE and MAE for validation and test datasets across number of neurons in (**a**) hidden layer #1, (**b**) hidden layer #2, (**c**) hidden layer #3 and (**d**) hidden layer #4.

As can be observed from the values in this table, the lower values of MSE and MAE and higher value of R^2^ are seen in higher epochs (10,000) regardless of the selected hidden layer. In contrast, the higher values of MSE and MAE and lower value of R^2^ are observed in lower epochs (100). These results show that the ANN model with 1000 epochs predicts more accurately than that with 100 epochs. Even though 10,000 epochs gives accurate results, selecting the number of hidden layers and number of neurons in each hidden layer at 10,000 epochs was obtained from a grid search. As the number of hidden layers was increased from 1 to 4 with a constant 10,000 epochs, MSE and MAE decreased, while R^2^ increased regardless of the number of neurons in the hidden layer (Table 2), and the optimal result (lower MAE and MSE, and higher R^2^) was observed in the case with 4 hidden layers. The next step was to compare the MAE, MSE, and R^2^ of the ANN model with 4 hidden layers and 10,000 epochs by varying the number of neurons in the hidden layers from 25 up to 200 (step size = 25). The variation in MAE and MSE across the number of neurons in hidden layers is shown in Figure 4. Based on the data in the figure, the ANN model with 4 hidden layers and 100 neurons in each hidden layer was selected as the best ANN architecture.

**Figure 5:**
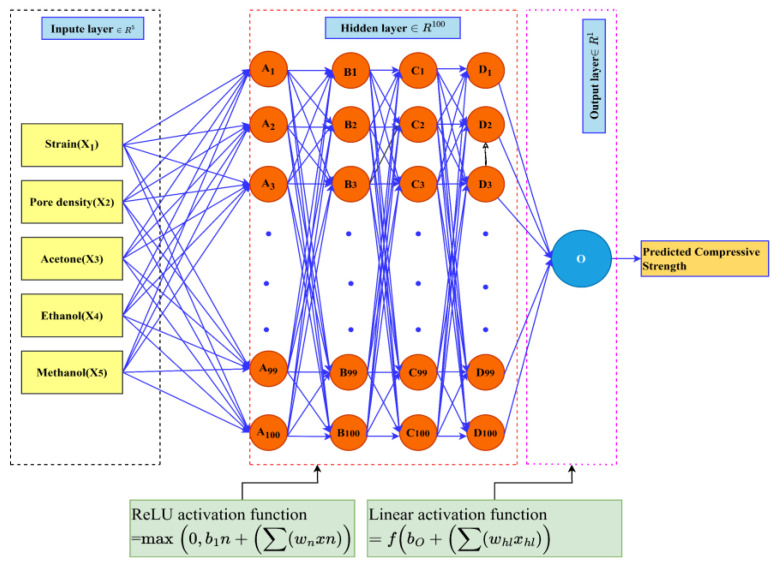
Architecture of backpropagation neural network.

The input parameters include strain, three pore density values (20, 40, and 60 ppi), and 3 solvents (acetone, ethanol, and methanol). Each solvent was used as separate input because categorical variables like ethanol, methanol, and acetone must be converted to binary form before training the ANN model. This method, known as one-hot encoding, transforms each category into a unique binary vector, allowing the ANN to interpret categorical data appropriately. Furthermore, the solvents were treated as categorical variables while the pore density contained numerical features. In this article, a multilayer perceptron neural network (MLPNN) model (Figure 5) was selected because the architecture provides universal approximators [65].

**Figure A1:**
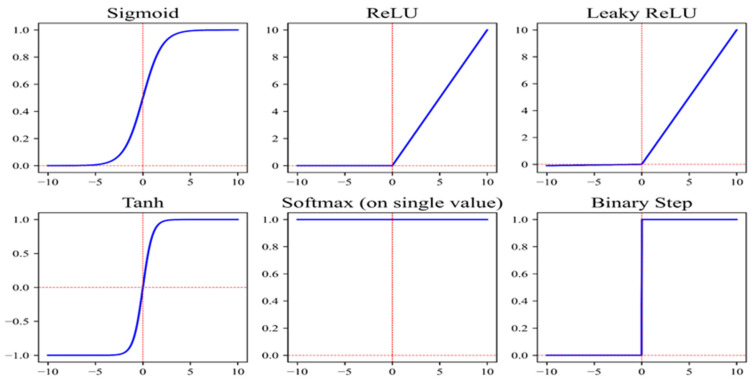
Types of activation functions.

Another important step in building an ANN is the choice of activation function, which depends on the problem and the network architecture [66]. A transfer function, or activation function, is a mathematical representation that is applied to the weights between layers, translating the input signals to the output signals [67]. Nowadays, the commonly used activation functions are sigmoid, SoftMax, tanh, ReLU, Leaky ReLU, and binary step [68]. Figure A1 in Appendix A shows various plots of typical activation functions. Among these, ReLU is currently the most used and appropriate for hidden layers of deep networks due to its computational efficiency, ability to address the vanishing gradient problem, and effectiveness in capturing complex global patterns, and in practice it converges six times faster than tanh and sigmoid [69]. For this reason, ReLU and linear activation function were selected for the hidden layers and the output layer and evaluated using Equations (2) and (3), respectively.

(2) $$ReLU\left(x\right)=\max\left(0,x\right)\;\;\;\;\;\;\;\;\;\;\;\;\;\;if\;x\geq 0,\;ReLU\left(x\right)=x,\;if\;x<0,\;ReLU\left(x\right)=0$$

(3) $$f\left(x\right)=x\;\;\;\;\;\;\;\;\;\;\;\;\;\;\;\;\;\;\;\;\;\;\;\;\;\;\;\;\;\;\;\;\;\;\;\;\;\;\;\;\;\;\;\;for\;all\;x,\;f\left(x\right)=x$$

**Figure 6:**
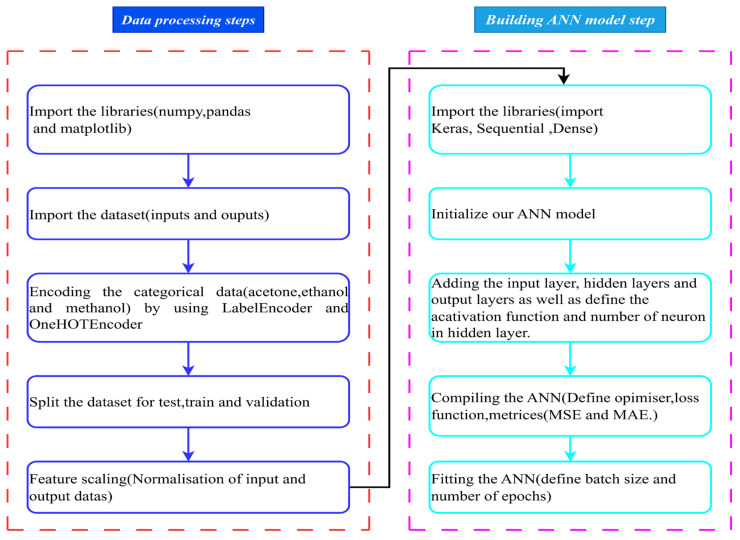
Keras TensorFlow steps to train ANN model.

The Adam optimizer with a default learning rate (0.001), 10,000 epochs, and batch size of 50 was used to train the model. The Adam optimizer is a widely used optimization algorithm for training neural networks and has advantages such as fast convergence and good adaptation to local minima issue [70]. Additionally, the Adam optimizer is known for its speed and stability, making it suitable for optimization problems with large datasets and high-dimensional spaces [71]. This optimizer updates parameters (weights and bias) by using Equations (4)–(9) [72]. On the other hand, the Keras neural network framework written in Python 3.13.1 was utilized to build and train the ANNs [73]. General steps for Keras tensor flow is shown in Figure 6.

(4) $$\mathrm{Gradient}\;\mathrm{of}\;\mathrm{the}\;\mathrm{loss}\;\mathrm{function}\;\;\;\;\;\;\;\;\;\;\;\;\;\;\;\;\;\;\;\;\;\;\;\;\;\;\;\;\;\;\;g_{t}=\nabla_{\theta }f(\theta_{t})$$

(5) $$\mathrm{Update}\;\mathrm{biased}\;\mathrm{moment}\;\mathrm{estimates}\;\;\;\;m_{t}=\beta_{1}m_{t-1}+\left(1-\beta_{1}\right)g_{t}$$

(6) $$\mathrm{Update}\;\mathrm{biased}\;\mathrm{moment}\;\mathrm{estimates}\;v_{t}=\beta_{2}v_{t-1}+\left(1-\beta_{2}\right)g_{t}^{2}$$

(7) Bias correction mt^=mt1−β1t

(8) Bias correction vt^=vt^1−β2t

(9) Parameter update                                              (θt+1)=θt−ηvt^+ϵ mt^

where $v_{t}$ is the exponential moving average of the gradient, $g_{t}$ is the first derivative for loss function (the gradient), η is learning rate, θ is parameters to optimize, and mt^ and vt^ are correction biases for $m_{t}$ and $v_{t}$, respectively. *β*_1_ and *β*_2_ are their exponential decay rate, and ϵ is a very small constant to avoid the denominator being zero (usually 10^−8^). The default values for β_1_ and β_2_ are 0.9 and 0.999, respectively. 

#### 2.2.3. Evaluation of ANN Model Performance

The performance of the ANN is evaluated and compared using several criteria, including MAE, RMSE, MSE, R^2^, and relative error between actual and predicted value [30,66], where the parameter *R*^2^ score explains the prediction strength against experimental observations in terms of a quantity ranging from 0 to 1. These parameters can be calculated as follows (Equations (10)–(14)):

(10) $$\mathrm{Mean}\;\mathrm{absolute}\;\mathrm{error}=\frac{1}{n}\sum_{i=1}^{n}\left|Y_{predicted}-Y_{actual}\right|$$

(11) $$\mathrm{Mean}\;\mathrm{Squared}\;\mathrm{Error}=\frac{1}{n}\sum_{i=1}^{n}{\left(Y_{predicted}-Y_{actual}\right)}^{2}$$

(12) $$\mathrm{Root}\;\mathrm{Mean}\;\mathrm{Squared}\;\mathrm{Error}=\sqrt{\frac{1}{n}\sum_{i=1}^{n}{\left(Y_{predicted}-Y_{actual}\right)}^{2}}$$

(13) $$\mathrm{Coefficient}\;\mathrm{of}\;\mathrm{Determination}=1-\frac{{\left(Y_{predicted}-Y_{actual}\right)}^{2}}{\sum_{i=1}^{n}{\left(Y_{predicted}-Y_{actual}\right)}^{2}}$$

(14) $$\mathrm{Relative}\;\mathrm{error}\;(\%)=\left(\frac{Y_{predicted}-Y_{actual}}{Y_{actual}}\right)\times 100$$

#### 2.2.4. Weight and Bias of the ANN Model

The weight and bias in the ANN are critical components that significantly influence the model’s performance and learning efficiency. The proper initialization and management of these parameters can enhance convergence speed and generalization capabilities [74]. In this article, the weight and bias were initialized by using default Keras and can be calculated by using the following equations.

From input to first hidden layer,

(15) $$z^{\left[1\right]}=W^{\left[1\right]}x+b^{\left[1\right]},\;A_{i}=ReLU\left(W^{\left[1\right]}x+b^{\left[1\right]}\right)=\max\left(0,W^{\left[1\right]}x+b^{\left[1\right]}\right)$$

From the first hidden layer to the 2nd hidden layer,

(16) $$z^{\left[2\right]}=W^{\left[2\right]}A+b^{\left[2\right]},\;B_{i}=ReLU\left(W^{\left[2\right]}A+b^{\left[2\right]}\right)=\max\left(0,W^{\left[2\right]}A+b^{\left[2\right]}\right)$$

From the 2nd hidden layer to the 3rd hidden layer,

(17) $$z^{\left[3\right]}=W^{\left[3\right]}B+b^{\left[3\right]},\;C_{i}=ReLU\left(W^{\left[3\right]}B+b^{\left[3\right]}\right)=\max\left(0,W^{\left[3\right]}B+b^{\left[3\right]}\right)$$

From the 3rd hidden layer to the 4th hidden layer,

(18) $$z^{\left[4\right]}=W^{\left[4\right]}C+b^{\left[4\right]},\;D_{i}=ReLU\left(W^{\left[4\right]}C+b^{\left[4\right]}\right)=\max\left(0,W^{\left[4\right]}C+b^{\left[4\right]}\right)$$

From the 4th hidden layer to the output layer,

(19) $$z^{\left[5\right]}=W^{\left[5\right]}D+b^{\left[5\right]},\;O=linear\left(W^{\left[5\right]}D+b^{\left[5\right]}\right)=W^{\left[5\right]}D+b^{\left[5\right]}$$

where $x={\left[X_{1},X_{2},X_{3},X_{4},X_{5}\right]}^{T},A_{i}={\left[A_{1},A_{2}\ldots A_{100}\right]}^{T},B_{i}={\left[B_{1},B_{2}\ldots B_{100}\right]}^{T},$ $C_{i}={\left[C_{1},C_{2}\ldots C_{100}\right]}^{T},D_{i}={\left[D_{1},D_{2}\ldots D_{100}\right]}^{T}\;and\;O=\left[O\right]$, and $W^{\left[1\right]}\in R^{100\times 5},W^{\left[2\right]}\in R^{100\times 100},W^{\left[3\right]}\in R^{100\times 100},W^{\left[4\right]}\in R^{100\times 100},\;and$ $W^{\left[5\right]}\in R^{1\times 100}$ is the weight matrix connecting the 5 input neurons to the 100 neurons in the first hidden layer, the weight matrix connecting the 100 neurons in the first hidden layer to the 100 neurons in the second hidden layer, weight matrix connecting the 100 neurons in the second hidden layer to the 100 neurons in the third hidden layer, the weight matrix connecting the 100 neurons in the third hidden layer to the 100 neurons in the fourth hidden layer, and the weight matrix connecting the 100 neurons in the fourth hidden layer to the output neuron, respectively. Additionally, $b^{\left[1\right]},b^{\left[2\right]},b^{\left[3\right]},b^{\left[4\right]},\;and\;b^{\left[5\right]}$ are the bias vector for the first, second, third, and fourth hidden layers and output layer, respectively. Generally, the hyperparameters used in this article are shown in Table 3.

## 3. Discussion of Results

**Figure 7:**
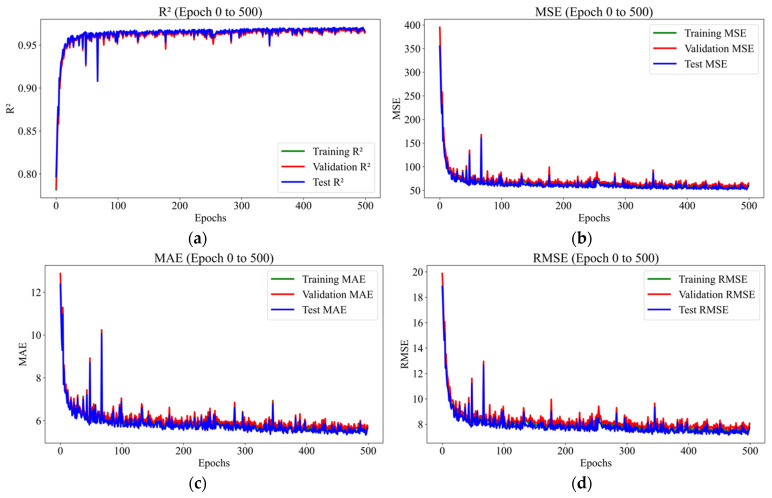
Variation of (**a**) R^2^, (**b**) MSE, (**c**) MAE, and (**d**) RMSE for selected epochs from 0–500.

**Figure 8:**
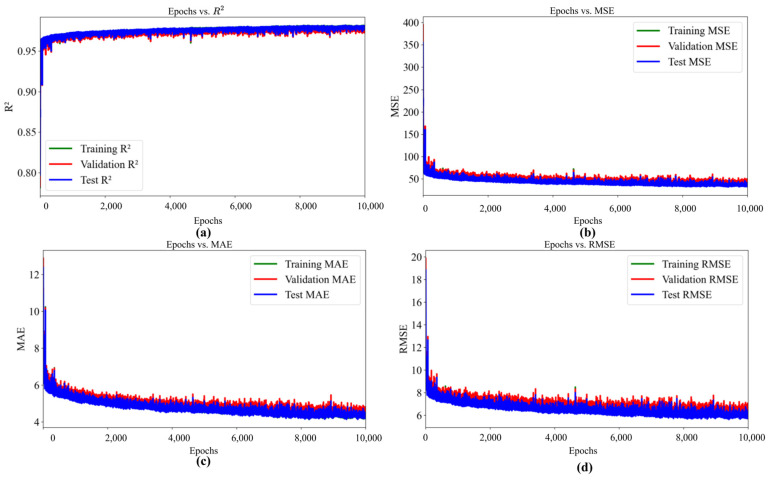
Variation of (**a**) R^2^, (**b**) MSE, (**c**) MAE, and (**d**) RMSE for epochs from 0–10,000.

The performance of the ANN during training over epochs, which was evaluated using R^2^, MSE, MAE, and RMSE for training, validation, and testing is shown in Figure 7. The R^2^ for training, validation, and test datasets are increased as the number of epochs increased from 0 to 500 (Figure 7a), whereas MSE, MAE, and RMSE decreased as the number of epochs increased from 0 to 500 (Figure 7b–d). This graph shows that the performance of the ANN is showing rapid improvement. A possible reason for this variation is due to the first epochs, the model parameters (weights and biases) are initialized randomly and are far from their optimal values. Additionally, in the early epochs, the gradients computed during backpropagation are relatively large because the model’s predictions are significantly different from the actual outputs. In contrast, as the number of epochs further increases and approaches 10,000 epochs, no significant changes in R^2^, MSE, MAE, and RMSE are observed (Figure 8a–d). The lack of significant changes in R^2^, MSE, MAE, and RMSE at higher epochs shows that the model has reached a state of convergence, where further training no longer provides meaningful improvements in performance. Additionally, model parameters (weights and biases) are no longer undergoing significant updates as the number of epochs increased above 10,000 epochs. Furthermore, the variation in R^2^, MAE, MSE, and RMSE for training, validation, and testing for all epochs are summarized in Table 4.

**Figure 9:**
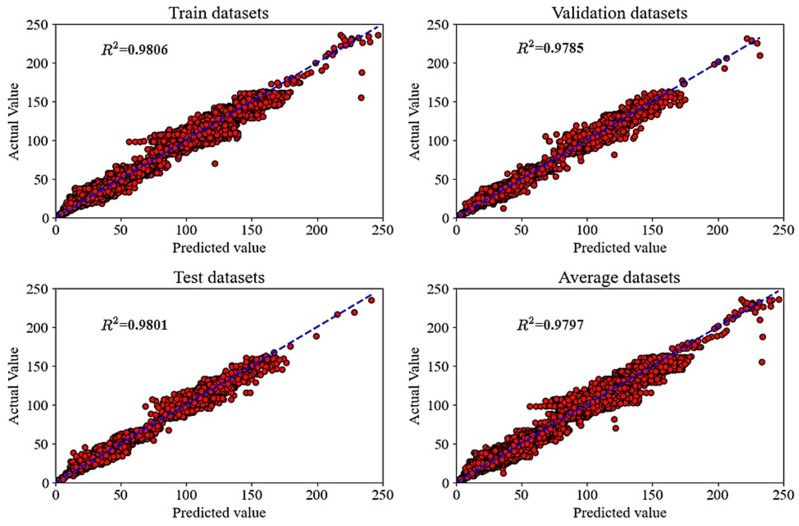
Regression of training, validation, testing, and average dataset.

Figure 9 shows the linear regression of actual (experimental) and predicted (ANN) values of compressive stress of carbon foam for various datasets (testing, training, and validation). The result shows that the R^2^ is close to 1 (0.9806, 0.9785, 0.9801, and 0.9797 for training, validation, testing, and average, respectively) for all datasets. This higher value of R^2^ in the linear regression (as shown by the blue trend lines in Figure 9) proves that the existence of a strong connection between the expected values and the observed experimental output. Additionally, the higher R^2^ values show excellent model performance with minimal variance across datasets, reflecting the strong predictive capability and generalization of the ANN. On the other hand, these results suggest that the ANN architecture (e.g., number of layers and neurons, activation functions) is well-suited for the problem.

**Figure 10:**
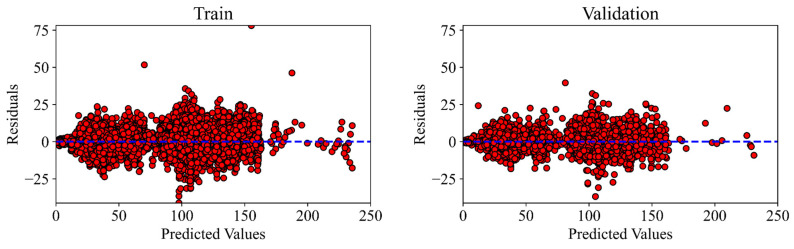

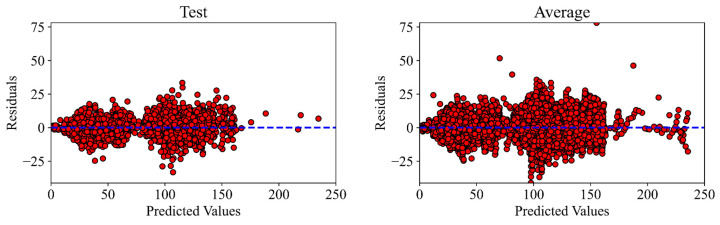
Residual plot for training, validation, testing, and average.

As shown in Figure 10, the average residual for training, validation, testing, and average datasets is 0.4484, 0.4999, 0.4923, and 0.4627, respectively. Residual values were calculated as the difference between the predicted and experimental (measured) data values for each data record in the entire dataset [75]. Moreover, from these figures, the residuals are spread evenly around the line y = 0 (as shown by the blue lines) without showing any visible pattern. This suggests that the ANN model is appropriately fit to the data structure without significant bias or variance issues.

**Figure 11:**
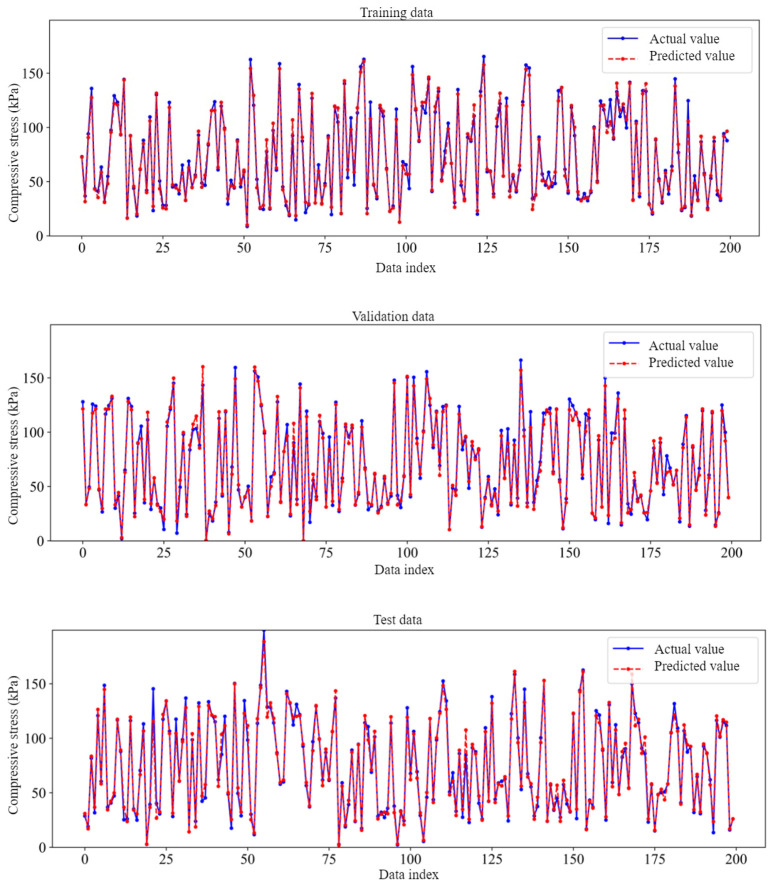
Comparisons between predicted and exact compressive stress values for 200 datasets.

Additionally, Figure 11 shows the direct comparison of experimental (actual) and ANN predicted compressive stress values for 200 data indexes for training, testing, and validation datasets. From this result, the experimental compressive stress values are almost the same as the ANN-predicted values with only small relative errors. This further supports that the BP neural network model has a high accuracy for predicting the compressive strength of carbon foam.

**Figure 12:**
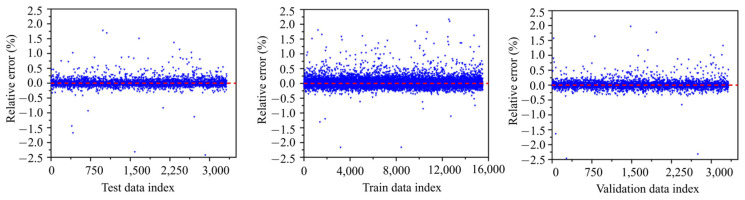
Relative errors (REs) of the predicted compressive stress values.

The relative error (difference between predicted and true values, divided by the true value) plot for testing, training and validation datasets versus data index are shown in Figure 12. For all datasets (testing, training, and validation), the relative errors are tightly centered around the red dotted line (zero error line). This indicates that ANN model prediction is close to experimental values. The errors are spread evenly across the indices for all datasets, with no systematic pattern (e.g., increasing or decreasing trends). This suggests that the model has well captured the underlying relationships in the data. As shown in Figure 12, the relative errors lay in the range ±2.5%. Among these, most relative errors lay in the range of ±0.5%. Higher relative errors (e.g., greater than ±0.5%) could result from noise or anomalies in the data, and limitations in the model’s ability to capture certain complex patterns. On the other hand, the similar error behavior across testing, training, and validation datasets highlights the ANN’s strong generalization ability.

**Figure 13:**
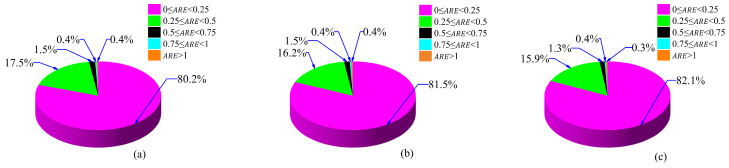
Proportion of errors across datasets for (**a**) validation, (**b**) testing, and (**c**) training.

Additionally, the variation in the absolute relative error (ARE) at five different levels is shown in Figure 13. The range of absolute relative errors lies within 0 to |2|%. These levels include (i) 0% ≤ ARE < 0.25%, (ii) 0.25% ≤ ARE < 0.5%, (iii) 0.5% ≤ ARE < 0.75%, (iv) 0.75% ≤ ARE < 1%, and (v) ARE > 1%. For the validation datasets around 80.2% (2655 data), 17.5% (579 data), 1.5% (50 data), 0.45% (15 data), and 0.36% (12 data), the absolute relative error is 0% ≤ ARE < 0.25%, 0.25% ≤ ARE < 0.5%, 0.5% ≤ ARE < 0.75%, 0.75 ≤ ARE < 1%, and ARE > 1%, respectively. For the test datasets, 81.4% (2694 data), 16.3% (540 data), 1.5% (50 data), 0.45% (15 data), and 0.39% (13 data) have an absolute relative error of 0% ≤ ARE < 0.25%, 0.25% ≤ ARE < 0.5%, 0.5% ≤ ARE < 0.75%, 0.75% ≤ ARE < 1%, and ARE > 1%, respectively. Lastly, for the training datasets, 82.1% (12,689 data), 15.9% (2458 data), 1.3% (201 data), 0.4% (62 data) and 0.32% (49 data) have an absolute relative error of 0% ≤ ARE < 0.25%, 0.25% ≤ ARE < 0.5%, 0.5% ≤ ARE < 0.75%, 0.75% ≤ ARE < 1%, and ARE > 1%, respectively. 

As shown in Table 5, the maximum ARE for the training, validation, and testing datasets is 1.95, 1.96, and 1.77, respectively. Conversely, the minimum error is 5.31 × 10^−6^, 6.18 × 10^−6^, and 0 for the training, validation, and testing datasets, respectively. The mean of the error is 8.16 × 10^−2^, 8.8 × 10^−2^, and 8.56 × 10^−2^ for the training, validation, and testing datasets, respectively. This result indicates that on average, the ANN-predicted values deviated by 8.16%, 8.8%, and 8.56% the from experimental values, respectively. Next, from the standard deviation values of the training, validation and testing datasets, the ANN-predicted compressive stress values deviated from the experimental values by 11.3, 12.2, and 12.1%, respectively. Finally, from the 95% CI, the results in this study show that the true mean error for the training, validation, and testing datasets lies in the range of 7.98 to 8.34%, 8.39 to 9.22%, and 8.17 to 9%, respectively. The lower range between the upper and lower 95% (0.0036 in the training, 0.0083 in the validation, and 0.0083 in the testing) in training, validation, and testing indicates a consistent ANN model performance and a sufficient sample size.

## 4. Conclusions

In this study, an ANN model was developed to predict the compressive properties of carbon foam based on various input parameters. The model was trained on historical data, and using multiple hidden layers and neurons, so that the ANN can capture complex relationships between the input features and the compressive stress values of the carbon foam. The network utilized the ReLU activation function in the hidden layers, which helped the model efficiently learn and represent non-linear relationships, while the output layer used a linear activation function to predict the continuous compressive strength values.

The results indicated that the ANN model has the potential to provide accurate predictions of compressive strength, offering valuable insights for the material design and optimization of carbon foams in industrial applications. The model can be further refined and tested on larger datasets to improve its generalization capability and predictive accuracy. By utilizing ANN-based modeling, significant improvements in the efficiency and cost-effectiveness of producing carbon foam materials can be achieved, potentially accelerating advancements in industries such as aerospace, automotive, and energy storage. Future work could explore the integration of additional features or the application of different ANN architectures, such as alternative deep learning models, to further enhance the prediction accuracy. Additionally, real-world data validation and testing will be essential to confirm the practical applicability of the model in predicting compressive strength under varying conditions and manufacturing processes.

## Figures and Tables

**Table 1:** Summary of reviewed articles relevant to this study.

No	Author(s)	Objectives	Materials	Methodology	Key Findings/Results
1	Zhuang et al. [41]	To predict mechanical properties of aluminum foam	Aluminum foam	2D convolutional neural network (2D-CNN) and conditional generative adversarial network (CGAN)	Achieved < 3% error in predicting mechanical properties of aluminum foam by using 2D-CNN.
2	Rodríguez-Sánchez et al. [42]	To map compressive stress response and energy absorption parameters of an expanded polystyrene foam	Expanded polystyrene foams	ANN	ANN model outperformed prediction capabilities of compressive strength and strain energy absorption of polystyrene foam and obtained errors around 2% of experimental data only.
3	Hangai et al. [43]	To estimate the plateau stress of aluminum foam	Aluminum foam	Supervised learning neural network model and X-ray computed tomography (CT)	Using an ANN is the most promising method and can train results obtained from advanced 3D imaging techniques such as CT-scan.
4	Hangai et al. [44]	To estimate the plateau stress of aluminum foam	Aluminum foam	CNN and X-ray CT	The plateau stresses estimated by machine learning and those obtained by the compression test were almost identical.
5	Rodríguez-Sánchez and Plascencia-Mora [45]	Predict the mechanical response of expanded polystyrene foam	Expanded polystyrene foams	Feed-forward ANN	ANN predicted the mechanical response almost the same with experimental values (errors of less than 3%).
6	Rodríguez-Sánchez and Plascencia-Mora [46]	Predict compressive stress responses of polymer foam by taking density, loading rate, and strain as input parameter	Expanded Polypropylene and expanded polystyrene foams	Feed-forwarded ANN with interpretability tool	Integration of interpretability tools with ANN models offers a robust method for material response analysis (compressive properties) and contributing to a deeper understanding of material science.
7	Stręk et al. [47]	Verify the possibility of describing compression phenomenon of closed-cell aluminum by ANNs	Closed-cell aluminum foams	ANNs and experimental	ANNs were found to be appropriate tools for building models of the compression phenomenon of aluminum foams.
8	Zhuang et al. [38]	To investigate the mechanical properties of Voronoi modeled aluminum foam	Aluminum foam	3D-CNN and FEA	Deep learning has more advantages in efficiency and accuracy of predicting mechanical properties of cellular solids and is an effective alternative to numerical simulation.
9	Ozan et al. [48]	To study effect of fabrication parameters on the pore concentration of aluminum metal foam	Aluminum foam	ANN and experimental	The ANN was successfully used to predict the pore concentration % (volume) of aluminum foam related to fabrication parameters.
10	Gahlen et al. [49]	To predict the orthotropic stiffness tensor of anisotropic foam structures utilizing a tessellation-based foam RVE database	Low-density closed-cell PUR	FEA and ANN	The anisotropy of complex foam structures can be determined via the ANN within seconds instead of performing time-consuming simulations (up to hours).
11	Pech-Mendoza et al. [50]	To predict the compressive stress responses of polystyrene foams	Expanded polystyrene	ANN	The utility of ANNs in modeling the compressive behavior of polystyrene foams resulted in errors of less than 3% as compared to the experiment.
12	Aengchuan et al. [51]	To predict the stress relaxation of polymer foam	Polymer foam	Feed-forward ANN	The results demonstrate that the ANN model achieved highly accurate predictions for the relaxation stress of polymer foam.
13	Dashtgoli et al. [52]	To investigate the mechanical behavior of biocomposite cellular sandwich structures under quasi-static out-of-plane compression	Bio-based cellular composite	Machine learning (ML)	Advanced ML models gave accurate predictions of the mechanical behavior of biocomposites, enabling more efficient and cost-effective development.
14	Abdellatief et al. [53]	To predict porosity and compressive strength of foam glass	Foam glass (FG)	Gradient boosting (GB), random forest (RF), gaussian process regression (GPR), and linear regression (LR)	The optimization of FG was production by providing reliable tools for predicting and controlling porosity and compressive strength, reducing material waste, enhancing product quality, and streamlining manufacturing processes.
15	Salami et al. [54]	To develop ANN, GEP, and GBT models for predicting compressive strength of foamed concrete	Foamed concrete	ANN, gene expression programming (GEP), and gradient boosting tree (GBT) models	A GBT model offered reliable accuracy in predicting the compressive strength of foamed concrete.

**Table 2:** Summary of grid search results.

Number of Hidden Layers	Epochs	Number of Neurons in Hidden Layers	Validation			Testing		
			MSE	R^2^	MAE	MSE	R^2^	MAE
1	100	150	158.16	0.9130	8.34	137.65	0.9210	7.98
	1000	125	117.10	0.9357	7.43	103.49	0.9409	7.15
	10,000	200	67.99	0.9630	6.19	64.19	0.9633	6.08
2	100	200	81.88	0.9550	6.44	72.47	0.9590	6.27
	1000	175	60.51	0.9668	5.72	54.88	0.9686	5.51
	10,000	200	49.69	0.9730	5.11	42.52	0.9760	4.86
3	100	125	68.57	0.9620	6.17	61.58	0.9650	5.92
	1000	200	53.15	0.9708	5.35	47.58	0.9728	5.14
	10,000	175	39.93	0.9780	4.62	36.44	0.9790	4.46
4	100	100	65.74	0.9640	5.99	61.22	0.9650	5.89
	1000	200	50.31	0.9724	5.21	45.47	0.9740	5.04
	10,000	100	39.10	0.9785	4.60	35.00	0.9801	4.40

**Table 3:** Summary of hyperparameters of the ANN model.

Parameters	Specification
ANN types	Feed-forward Neural Networks
Loss function	Mean square error
Optimizer	Adam
Number of neurons in input layer	5
Number of hidden layers	4
Number of neurons in hidden layers	100
Number of neurons in output layer	1
Activation function in hidden layer	ReLU
Activation function in output layer	Linear
Input	Strain, pore density, and solvents
Output	Compressive stress

**Table 4:** Performance of the developed ANN model.

Training				Validation Data				Test			
MSE	MAE	RMSE	R^2^	MSE	MAE	RMSE	R^2^	MSE	MAE	RMSE	R^2^
34.93	4.28	5.89	0.9806	39.1	4.6	6.22	0.9785	35	4.4	5.89	0.9801

**Table 5:** Descriptive statistics of ARE for training, validation, and testing datasets.

Statical Parameters	Training Datasets	Validation Datasets	Testing Datasets
Minimum Error	5.31 × 10^−6^	6.18 × 10^−6^	0
Maximum Error	1.95	1.96	1.77
Mean	8.16 × 10^−2^	8.8 × 10^−2^	8.58 × 10^−2^
Standard Deviation	1.13 × 10^−1^	1.22 × 10^−1^	1.21 × 10^−1^
Lower 95% CI of mean	7.98 × 10^−2^	8.39 × 10^−2^	8.17 × 10^−2^
Upper 95% CI of mean	8.34 × 10^−2^	9.22 × 10^−2^	9 × 10^−2^

## Data Availability

The raw data supporting the conclusions of this article will be made available by the authors on request.
